# Partial-mouth plaque recording revisited: evaluation of tooth- and surface-based subsets using a data-driven benchmark

**DOI:** 10.1007/s00784-026-07024-1

**Published:** 2026-07-16

**Authors:** Katja Jung, Franziska Eilert, Philipp Hewing, Christina Hölbling, Carolina Ganss

**Affiliations:** 1 Present Address: Marburg University, University Dental Medicine, Clinic for Operative Dentistry, Endodontics, and Pediatric Dentistry, Section for Cariology, Marburg, Germany; 2 Dental Practice, Donzdorf, Germany; 3 Dental Practice, Wetzlar, Germany; 4 Dental Practice, Lörrach, Germany

**Keywords:** Partial-mouth recording, Dental plaque, Plaque indices, Planimetric plaque assessment, Oral hygiene, Preventive dentistry, Intraoral scanning

## Abstract

**Objectives:**

To evaluate the validity of established and newly developed partial-mouth recording subsets for approximating full-mouth plaque across different plaque metrics and oral hygiene states, and to determine whether surface-based subsets derived from the observed plaque distribution offer advantages over established recording schemes.

**Materials and methods:**

Disclosed-plaque intraoral scans from 30 adults (24.0 ± 4.1 years) were obtained at baseline, after 72 h plaque accumulation, and after toothbrushing. Plaque was quantified planimetrically (percentage plaque coverage, P%) and using two plaque indices (Turesky modified Quigley–Hein Plaque Index (TQHPI); Rustogi modified Navy Plaque Index (RMNPI)). Established Ramfjord (6 teeth/12 surfaces) and Community-Periodontal-Index-of-Treatment-Needs (CPITN; 6 teeth/12 surfaces or 10 teeth/20 surfaces) subsets, optimised subsets derived from the observed plaque distribution to represent overall plaque levels, and additional subsets representing plaque-prone sites with elevated plaque levels were compared with full-mouth recordings. Performance was assessed using bias, root-mean-square error, intraclass correlation coefficients (ICC), and mixed-effects modelling.

**Results:**

An Optimised-12 surface subset showed near-perfect agreement with P% full-mouth recordings (bias 0.03, ICC 0.962); the 12-surface CPITN revealed similar results (bias -0.07, ICC 0.967). The Ramfjord subset showed slight underestimation but high agreement (bias -1.23, ICC 0.970), whereas the 20-surface CPITN consistently overestimated full-mouth plaque (bias 4.33, ICC 0.839). A reduced 6-surface Ramfjord subset also preserved high agreement with full-mouth recordings (bias -0.76, ICC 0.954). For surfaces with elevated plaque levels, newly developed 12- and 6-surface subsets closely matched the elevated-plaque reference. Subset performance patterns were consistent across all oral hygiene stages and across plaque metrics (P%, TQHPI, RMNPI).

**Conclusions:**

Except for the 20-surface CPITN, all subsets provided a robust approximation of full-mouth plaque. Surface-based subsets derived from the observed plaque distribution offer accurate alternatives for assessing overall plaque, while the elevated-plaque subsets provided a useful approach for monitoring plaque-prone sites.

**Clinical relevance:**

Time-efficient partial-mouth recording may support feasible plaque monitoring in clinical research and preventive care, particularly when full-mouth assessment is impractical.

**Supplementary Information:**

The online version contains supplementary material available at 10.1007/s00784-026-07024-1.

## Introduction

Dental plaque is the modifiable ecological cause of the most prevalent oral diseases, including caries, gingivitis, and periodontitis [1–4]. Reliable assessment of plaque levels is therefore fundamental to both clinical research and preventive dentistry. While full-mouth plaque recording is commonly regarded as the reference standard, it is time-consuming, examiner-intensive, and often impractical in large studies or repeated assessments. These limitations have led to the development of partial-mouth recording protocols [5, 6]. In addition to research settings, reliable partial-mouth recording could support feasible plaque monitoring in routine preventive care, where chairside time is limited but repeated assessment may be valuable for documenting oral hygiene status and response to instruction over recall visits.

Historically, partial-mouth plaque recording has focused almost exclusively on the selection of teeth rather than tooth-surface combinations. Classic protocols, such as the Ramfjord index teeth [5] or Community Periodontal Index of Treatment Needs (CPITN) subsets [6], were primarily developed to maximise feasibility and standardisation in epidemiological surveys. Their selection was guided by pragmatic considerations rather than by empirical evidence on plaque distribution within the dentition. Implicitly, if used for plaque recording, these approaches assume that selected teeth adequately represent the overall plaque burden, and that individual tooth surfaces contribute proportionally to this representation.

However, available evidence indicates that plaque distribution within the dentition is neither uniform nor random. Distinct spatial gradients have been described, with higher plaque accumulation in posterior compared with anterior regions, differences between maxillary and mandibular arches, and, most importantly, systematic variation between oral (i.e. lingual and palatal) and vestibular (i.e. buccal and facial) surfaces [7, 8]. Moreover, plaque accumulation exhibits reproducible, individual-specific patterns over time, suggesting that certain teeth and surfaces consistently function as plaque-prone sites [9]. Together, these findings challenge the assumption that teeth are interchangeable sampling units and indicate that plaque-related information is concentrated in specific tooth-surface locations.

Despite the central role of plaque distribution in plaque-associated diseases, systematic investigations of tooth- and surface-specific plaque patterns remain surprisingly limited. This gap is largely attributable to the historical reliance on plaque indices that intentionally collapse spatial information into summary scores, such as the Turesky modified Quigley-Hein Plaque Index (TQHPI) [10] and the Rustogi modified Navy Plaque Index (RMNPI) [11]. While such indices are well suited for comparing individuals or populations, they obscure within-mouth heterogeneity and restrict insight into where plaque accumulates and how these patterns change. As a result, much of the spatial information inherently contained in plaque data is lost by design. This reduction is compounded by the fact that ordinal plaque index scores are statistically difficult to interpret, because their categories do not represent proportional differences in plaque amount. Likewise, summarising plaque as a whole-mouth mean may further obscure within-mouth heterogeneity by masking local plaque-prone sites and site-specific changes over time.

Notably, plaque levels can be quantified using conceptually different measurement approaches. Surface-based planimetric [12–15] or volumetric [16] methods quantify the extent or thickness of plaque as a continuous variable, whereas commonly used plaque indices rely on ordinal or semi-quantitative scoring systems that emphasise the presence and coverage of plaque on predefined surface regions. Although these approaches are correlated, they capture distinct aspects of the plaque construct and may differ in their sensitivity to high versus low plaque levels, as well as to temporal changes following plaque accumulation or removal [17].

This distinction has direct implications for partial-mouth recording strategies. The suitability of a given tooth or surface subset may depend not only on its representativeness for mean plaque levels, but also on how plaque is operationalised by the measurement method. Ordinal indices may be disproportionately influenced by plaque-prone surfaces, whereas continuous planimetric measures may reflect more diffuse plaque accumulation across the dentition. Whether partial-mouth subsets perform consistently across different plaque metrics, and whether they reliably capture transitions between low and high plaque states, remains unclear.

Although numerous partial recording protocols have been evaluated against full-mouth scores for assessing periodontitis [18], partial mouth recordings have been much less evaluated for plaque recording [19–23]. Moreover, the suitability of partial-mouth recordings for monitoring plaque dynamics, including plaque growth and post-brushing effects, has not been systematically assessed. This gap is particularly relevant given that plaque accumulation and removal are surface-specific processes and that the sensitivity of different plaque metrics to change may vary across surfaces and plaque levels. Consequently, cross-sectional validity does not necessarily imply longitudinal validity across different measurement constructs.

Against this background, the present study evaluates existing tooth-based partial-mouth recording protocols and, in parallel, explores the development of data-driven, surface-based subsets derived from observed plaque distribution patterns in the present dataset. In this context, “data-driven” means that tooth-surface combinations were selected according to their empirical ability to represent full-mouth plaque levels, rather than being chosen solely on the basis of established index-tooth concepts. The resulting best-performing combinations were used as empirically optimised benchmark subsets. By comparing these subsets with established recording schemes and assessing performance across both planimetric and index-based plaque measures, this study investigates whether partial-mouth recording can validly represent full-mouth plaque levels and their temporal changes, including plaque accumulation and post-brushing effects. In doing so, it aims to contribute to a more biologically and measurement-informed framework for partial-mouth plaque assessment in both research and preventive care contexts.

## Materials and methods

The study is a secondary analysis of pseudonymised intraoral scans obtained from an earlier study [17] which was conducted at the Department of Restorative Dentistry and Endodontology, Dental Clinic, Justus-Liebig-University Giessen, Germany, approved by the local Ethics Committee of the Medical Faculty of Giessen (ref. no. 61/22) and recorded in the German Clinical Trial Register (DRKS00033908). The study was conducted in accordance with good clinical practice and the Declaration of Helsinki. The secondary analysis was also approved by the Ethics Committee of the Medical Faculty of Giessen (Amendment to ref. no. 61/22).

In brief, thirty participants (19 females, 11 males; mean age 24.0 ± 4.1 years) were included. Participants were students of our university and recruited via standard university information channels. Inclusion criteria were age 18 years or older, written informed consent, complete dentition (except third molars) without restorations on vestibular and oral smooth surfaces beyond the extent of pit and fissure sealants on the surfaces of interest. Exclusion criteria were intolerance to the materials used (Mira-2-Ton® solution: sodium benzoate, potassium sorbate, C. 45,410, C. 42,090; OptraGate lip retractor: styrene-ethylene-butylene-styrene), cavitated caries, gap teeth and gingival recession of more than 1/3 of the root length, fixed orthodontic appliances (retainers allowed), and physical or mental limitations of any kind that could affect performance.

Intraoral scans (Dexis IS CS 3800 W, DEXIS, Atlanta, GA, USA) were performed at baseline T1 (habitual plaque), at T2 (after 72 h refraining from oral hygiene) and at T3 (after toothbrushing performed to the best of the participants’ ability, without standardized instructions regarding brushing duration, systematic approach, or technique). Plaque was disclosed with a plaque revelator (Mira-2-Ton®, Hager & Werken GmbH & Co KG, Duisburg, Germany), applied twice with a saturated foam pellet. After each application, participants rinsed with water for 10 s. During scanning, a cheek retractor (OptraGate; Ivoclar Vivadent. Schaan. Liechtenstein), parotid patches (DryTips®, Microbrush, Algonquin, IL, USA) and a saliva ejector were used to keep the dentition dry.

### Scan alignment

The intraoral scans were exported as polygon (PLY) files and processed in MeshLab. For each participant and jaw, the three scans (T1, T2 and T3) were aligned using several landmarks on reproducible locations of the tooth crowns in each scan. The three aligned scans were then manually rotated simultaneously about the x-, y-, and z-axes, maintaining identical orientation across scans, until an orthograde view of the tooth surface of interest was obtained, with the mesial and distal aspects equally visible. PNG screenshots were subsequently generated from all aligned scans simultaneously, after which the oral or vestibular surfaces of each tooth were cropped and saved as separate files against a black background.This workflow produced a standardized orientation across all three time points, ensuring that the same tooth-surface regions can be evaluated consistently.

### Plaque assessment

Plaque amounts were quantified with planimetry and with two plaque indices on oral and vestibular surfaces of all teeth except third molars.

For the planimetric evaluation [15], tooth surfaces of interest were cropped from the screenshots, each placed on a black background and saved as.jpg files. The images were analysed using an algorithm obtained from the Julia programming language and the “ImageMagick” and “Images” packages. After conversion in an RGB image, the red, green, and blue values of each pixel were determined, transformed into a 3-component vector system, and stored separately. Two filters were then applied to calculate plaque coverage (P%): the first excluded all black background pixels (RGB = 0,0,0), while the second applied a threshold to differentiate between pixels representing plaque-covered areas and those corresponding to plaque-free tooth surfaces. Plaque coverage (P%) was quantified using standardised filters and threshold settings to distinguish plaque-covered from plaque-free areas. Batch processing allowed for the automatic calculation and export of P% values. The output images were visually compared with the original images and re-quantified in cases of substantial misclassification.

Plaque indices were the Turesky modified Quigley and Hein Plaque Index (TQHPI) [10], and the Rustogi modified Navy Plaque Index (RMNPI) [11].

For standardisation, a grid representing the RMNPI and TQHPI areas was projected onto and adjusted to a given tooth surface. For the RMNPI, each of 9 areas was assessed for plaque containing or plaque free, for the TQHPI the grid was used to standardise the grades 3 to 5.

### Reproducibility

For planimetric plaque quantification, the planimetrically assessed plaque data obtained by F.E. on the Ramfjord teeth (16, 21, 24, 36, 41, and 44) were compared with the planimetric data obtained by C.H. The analysis was based on three participants, each assessed at three time points. Scan orientation, alignment, cropping, and plaque quantification were performed independently by the examiners, with an interval of several months between assessments and without mutual coordination. Despite this, reproducibility was very high: the ICC was 0.945 [0.902–0.974] at T1, 0.943 [0.901–0.973] at T2, and 0.905 [0.833–0.954] at T3.

C.H.’s intrarater calibration across all teeth for three participants yielded ICC values of 0.966 [0.935–0.983] at T1, 0.983 [0.966–0.991] at T2, and 0.983 [0.967–0.991] at T3.

Plaque indices (RMNPI and TQHPI) were scored by P.H., who underwent intensive training prior to study start. Inter-rater reliability between P.H. and F.E. was assessed using unaggregated weighted Cohen’s kappa on a Ramfjord-tooth subset from three participants across all three time points. Weighted kappa values for the TQHPI were 0.88 (T1), 0.89 (T2), and 0.87 (T3), and for the RMNPI 0.80 (T1), 0.76 (T2), and 0.79 (T3) (all p < 0.001).

To assess intra-rater reliability, P.H. repeated the scorings after three weeks, resulting in weighted kappa values of 0.92 (T1), 1.00 (T2), and 0.97 (T3) for the TQHPI, and 0.90 (T1), 0.94 (T2), and 0.92 (T3) for the RMNPI (all p < 0.001).

### Partial mouth recording subsets

#### Subsets for overall plaque representation

Empirically derived (data-driven) subsets of tooth-surface combinations optimised for representing overall plaque levels were generated from the full-mouth data. Two subset sizes were evaluated, comprising 12 (Optimised-12) and 6 (Optimised-6) tooth-surface combinations. The Ramfjord subset comprised the original six index teeth (16, 21, 24, 36, 41, 44). For each tooth, both the oral (o) and vestibular (v) surfaces were included, resulting in 12 tooth-surface combinations (Ramfjord-12). From this set, a reduced Ramfjord-based subset comprising 6 tooth-surface combinations was also obtained (Ramfjord-6). Two CPITN tooth subsets [19] were analysed, comprising 10 teeth (17, 16, 11, 26, 27, 37, 36, 31, 46, 47) and 6 teeth (16, 11, 26, 36, 31, 46). For each tooth included, both the oral and vestibular surfaces were recorded, yielding 20 and 12 tooth-surface combinations, respectively (CPITN-20 and CPITN-12 resp).

#### Subsets for elevated plaque levels

In addition, separate 6- and 12-surface subsets were generated to represent tooth-surface combinations exhibiting comparatively elevated plaque levels across the dentition, that is, plaque-prone sites rather than overall full-mouth plaque (Elevated-12 and Elevated-6 resp.).

Details of the procedures used to identify the empirically derived subsets are provided in the Statistical Analysis section.

### Statistical analysis

All analyses were performed in a local Spyder (version 5.4.5) environment using Python 3.10. Core analyses relied on standard scientific libraries including pandas and numpy for data handling, scipy for statistical testing, pingouin for intraclass correlation coefficients, and statsmodels for mixed-effects modelling. Custom Python scripts were used throughout to ensure reproducibility.

All data are presented as mean P% ± standard deviation (SD).

### Optimisation of 6- and 12-surface subsets to approximate full-mouth plaque

To evaluate whether reduced sets of tooth surfaces can approximate full-mouth plaque measurements, a data-driven subset selection was performed using P% values at baseline (T1), after plaque accumulation (T2), and after brushing (T3). For each tooth-surface combination, correlations with full-mouth plaque values were calculated across all subjects and time points, and the 20 surfaces with the highest mean correlations were retained. From these, all possible 12 and 6 tooth-surface subsets were generated and filtered using predefined anatomical constraints to ensure balanced representation. For each subset, subject-level plaque values were calculated by averaging across selected surfaces, and performance across T1 to T3 was quantified using bias, root mean square error (RMSE), and Pearson correlation. For each subset size, the configuration with the lowest mean RMSE across time points was selected to define the final subsets (Optimised-12 and Optimised-6). Because the Optimised-6 subset was anatomically complex and impractical for routine use, a secondary optimisation was performed within the Ramfjord teeth [5]. All possible six-surface combinations derived from the Ramfjord index were evaluated using the same metrics, and the subset with the lowest mean RMSE was selected as the optimal Ramfjord-6 configuration (Ramfjord-6). In addition, the two versions of the CPITN subsets [19] were included. Subject-level means were calculated for P% and each index and phase (T1, T2, T3) for full-mouth and subsets.

### Performance metrics for subset agreement with full-mouth recordings

Agreement of subsets with full-mouth recordings was assessed using bias, RMSE, RMSE normalised to the full-mouth mean (RMSE%), intraclass correlation coefficients (ICC(2,1), absolute agreement), and Wilcoxon signed-rank tests.

In addition, linear mixed-effects models were fitted to assess the relationship between subset-based and full-mouth plaque values across time. For each index and subset, models included full-mouth plaque values and measurement phase as fixed effects and subject as a random intercept. Fixed-effect slopes and intercepts were used to evaluate proportional tracking and systematic offsets, and variance components were examined to compare residual variability between subsets.

### Composite scoring and evaluation of elevated plaque-level subsets

Elevated plaque level sites were identified at the level of individual tooth-surface combinations using P% data. For each subject, plaque values at T1, T2 and T3 were normalised by subtracting the subject-specific mean. Mean normalised deviations across subjects were calculated for each phase, standardised, and combined into a composite score. Tooth-surface combinations were ranked according to this score, and the 12 highest-ranking sites were used to define data-driven elevated plaque level subset reference (Elevated-ref). This subset was modified for clinical use with respect to symmetry in a 12- and 6-surface subset (Elevated-12 and Elevated-6).

To assess whether the Elevated-12 and Elevated-6 subsets preserve elevated-plaque level information beyond established tooth-based recording schemes, the elevated plaque level subsets were compared with CPITN subsets. Agreement with the reference was assessed using bias, RMSE, Pearson correlation, ICC(2,1), and Wilcoxon signed-rank tests, calculated for individual time points and pooled data.

## Results

### Plaque distribution pattern across the dentition

**Fig. 1:**
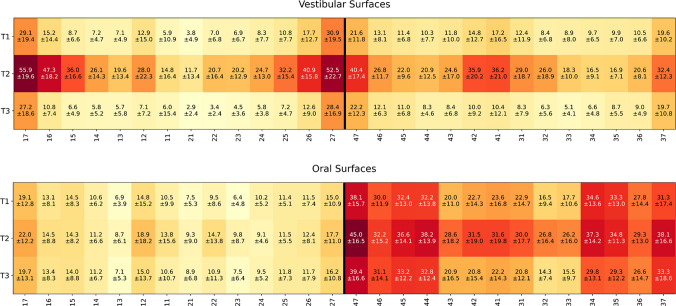
Heatmaps showing the plaque distribution pattern across the dentition (values inside cells: mean P% ± SD) at each tooth for vestibular (upper panel) and oral (lower panel) surfaces at the three time points: baseline (T1), plaque accumulation (T2), and post-brushing (T3)

Plaque distribution patterns (P%) across the dentition at T1, T2, and T3 are shown in the heatmaps (Fig. 1). At baseline (T1), vestibular surfaces generally exhibited low to moderate plaque levels, with the highest values observed on posterior sites, particularly on the maxillary second molars. Oral surfaces displayed a markedly different pattern: maxillary oral sites showed a relatively even distribution of low to moderate plaque accumulation, whereas mandibular oral surfaces demonstrated consistently high plaque levels, most pronounced on premolars and molars.

Following plaque accumulation (T2), vestibular surfaces of all teeth showed a distinct overall increase, maintaining the same spatial distribution pattern as at T1. Oral surfaces, in contrast, showed almost no increase on maxillary sites and only a moderate rise on mandibular posterior sites, preserving the characteristic mandibular hotspot pattern.

After brushing (T3), plaque levels decreased across nearly all surfaces, largely returning to the magnitudes and spatial distribution observed at baseline.

### Full mouth versus partial plaque recording

**Fig. 2:**
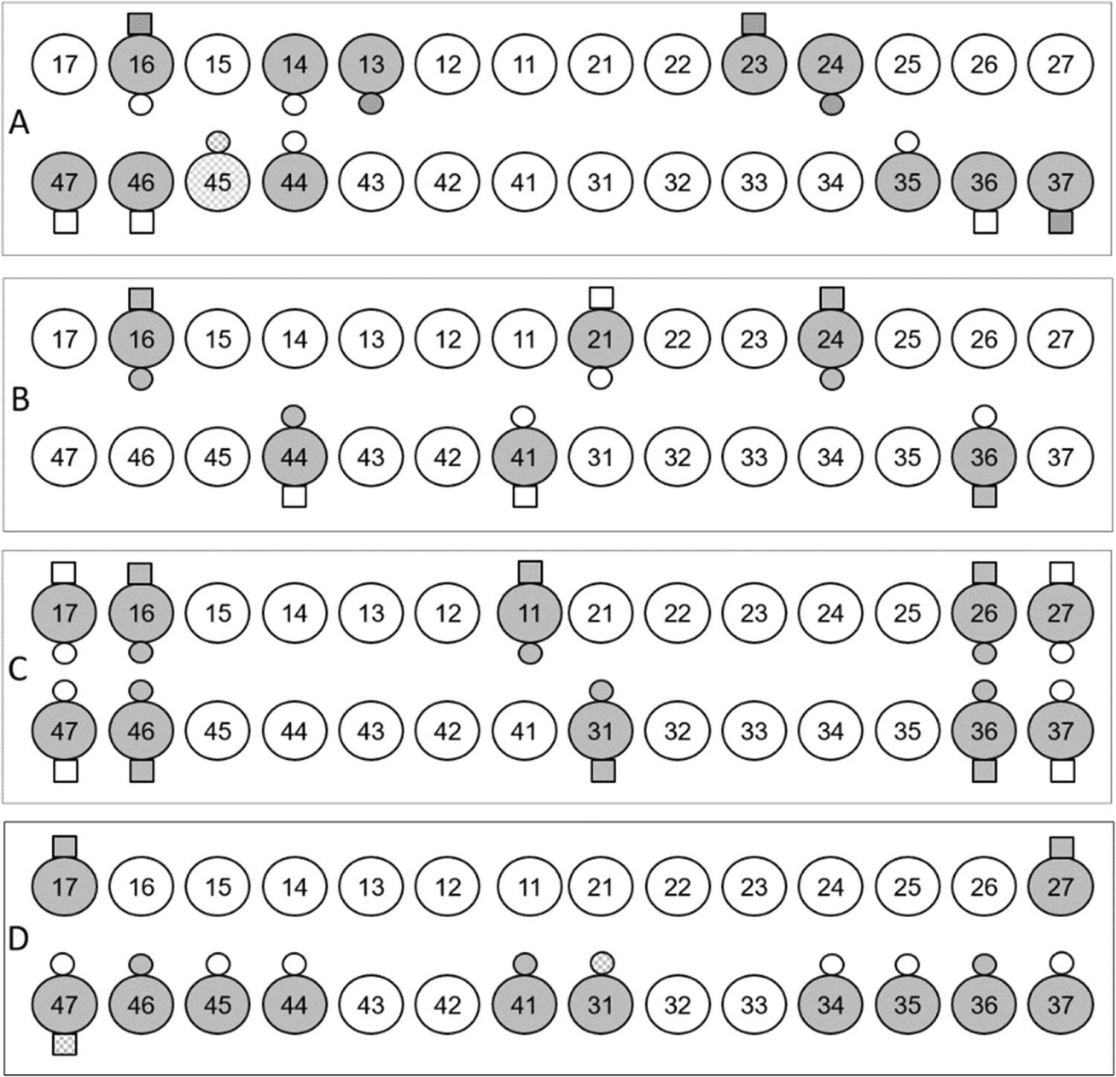
Schematic representation of the evaluated partial-mouth recording subsets. Markers indicate included tooth-surface combinations (squares: vestibular surfaces; circles: oral surfaces); surface designation follows the schematic orientation, with markers placed towards the oral cavity (between the arches) representing oral surfaces and markers placed towards the vestibule (outside the arches) representing vestibular surfaces. White markers and grey markers combined indicate the 12-surface subsets, grey markers denote the corresponding six-surface subsets. Panels show (A) empirically optimised subsets (optimised-6 or optimised-12), (B) Ramfjord and Ramfjord-based subsets, (C) CPITN subsets, and (D) subsets representing tooth-surface combinations with elevated plaque levels. For the optimised subsets, the teeth included in Optimised-6 correspond to those in Optimised-12, except for tooth 45 in panel A, which is indicated by a checkerboard pattern. For the elevated-plaque subsets (panel C), one surface was exchanged (31 o was added and 47 v was removed) to maintain symmetry; the affected markers are indicated by a checkerboard pattern

The search for reduced subsets that best approximated full-mouth P% values identified the following combinations: a 12-surface subset comprising 16-o, 16-v, 14-o, 13-o, 23-v, 24-o, 37-v, 36-v, 35-o, 44-o, 46-v, and 47-v (Optimised-12), and a 6-surface subset comprising 16-v, 13-o, 23-v, 24-o, 37-v, and 45-o (Optimised-6). The best approximated reduced Ramfjord-based alternative was 16-o, 16-v, 24-o, 24-v, 36-v, and 44-o (Ramfjord-6) (Fig. 2).

When plaque levels at all three time points were analysed jointly, a consistent pattern emerged across all approaches (P%, TQHPI and RMNPI, Table 1). The Optimised-12 subset showed the closest alignment with full-mouth recordings. Its mean values were virtually indistinguishable from the full-mouth scores, and agreement metrics indicated both very small deviations and highly stable correspondence. Statistical comparisons revealed no systematic differences to the full-mouth plaque levels.

**Table 1:** Performance of subsets relative to full-mouth plaque assessments across merged time points (T1–T3) for P%, TQHPI and RMNPI. RMSE: root-mean-square error, RMSE%: RMSE/full-mouth*100, ICC(2,1): two-way random-effects intraclass correlation for absolute agreement. The p value refers to the comparison of subsets to the full mouth plaque level

	Mean ± SD	Bias	RMSE	RMSE%	ICC(2,1)	p value
**P%**						
Full-mouth	18.8 ± 8.2					
Optimised-12	18.9 ± 8.1	0.03	2.24	11.91	0.962	0.534
Ramfjord-12	17.6 ± 8.1	−1.23	2.01	10.66	0.970	< 0.001
Optimised-6	18.3 ± 8.6	−0.49	2.41	12.77	0.959	0.045
Ramfjord-6	18.1 ± 8.8	−0.76	2.58	13.69	0.954	0.004
CPITN-20	23.3 ± 9.7	4.33	5.39	28.62	0.839	< 0.001
CPITN-12	18.8 ± 8.9	−0.07	2.19	11.61	0.967	0.406
**TQHPI**						
Full-mouth	2.6 ± 0.6					
Optimised-12	2.6 ± 0.6	−0.01	0.20	7.87	0.938	0.428
Ramfjord-12	2.4 ± 0.6	−0.17	0.27	10.33	0.903	< 0.001
Optimised-6	2.5 ± 0.6	−0.08	0.25	9.85	0.913	0.007
Ramfjord-6	2.5 ± 0.7	−0.10	0.37	14.41	0.833	0.016
CPITN-20	2.8 ± 0.6	0.22	0.31	12.01	0.869	< 0.001
CPITN-12	2.6 ± 0.6	0.00	0.21	8.03	0.938	0.873
**RMNPI**						
Full-mouth	6.3 ± 1.1					
Optimised-12	6.3 ± 1.1	−0.01	0.43	6.78	0.921	0.795
Ramfjord-12	6.2 ± 1.1	−0.05	0.50	7.97	0.895	0.057
Optimised-6	6.1 ± 1.2	−0.20	0.50	7.90	0.905	< 0.001
Ramfjord-6	6.2 ± 1.2	−0.10	0.70	11.09	0.814	0.045
CPITN-20	6.8 ± 1.0	0.48	0.60	9.60	0.851	< 0.001
CPITN-12	6.5 ± 1.1	0.17	0.47	7.40	0.912	< 0.001

The Ramfjord-12 subset also performed reasonably well, showing good overall agreement but with a slight tendency to underestimate plaque and with indications of a small systematic shift. Among the alternative 12-surface reference sets, the CPITN-12 subset yielded results that were very similar to those of the Optimised-12 subset, with minimal bias, low RMSE values and high agreement, closely approximating full-mouth plaque levels across all three plaque measures.

In contrast, the CPITN-20 subset showed a consistent tendency towards higher plaque values compared with full-mouth recordings. This was reflected by a systematic positive bias, larger RMSE values and reduced agreement metrics across all three plaque indices, indicating an overall overestimation of full-mouth plaque levels.

Among the 6-surface subsets, the Optimised-6 subset reproduced full-mouth levels more reliably than the Ramfjord-6 surface set. Although both showed modest discrepancies, the Optimised-6 subset demonstrated better precision and stronger agreement. In contrast, the Ramfjord-6 subset consistently showed the weakest performance, with noticeable underestimation, larger errors and reduced agreement metrics.

Analyses at the individual time points (T1, T2 and T3) showed the same pattern as the merged results: the optimised subsets—particularly the 12-surface version—consistently aligned most closely with full-mouth scores, whereas the Ramfjord subsets showed larger deviations. CPITN-12 again closely matched full-mouth recordings at all time points, while CPITN-20 consistently yielded higher plaque estimates. No additional or time-specific differences emerged, and the complete results are provided in the Supplementary Table S1.

The mixed-effects analyses showed that the Optimised-12 subset exhibited near-perfect proportional tracking of all three full-mouth plaque measures, with minimal constant offset, very small phase-specific effects, and low between- and within-subject variability. Most other subsets showed comparable tracking performance, with residual variability that was small relative to the scale of the respective plaque measures, indicating good precision of partial-mouth recording across indices. An exception was the Ramfjord-12 subset when applied to the TQHPI: although changes in TQHPI were tracked very closely and precisely, the model indicated a moderate systematic offset (constant underestimation) relative to full-mouth TQHPI. A similar pattern was observed for CPITN-20, but in the opposite direction, with a moderate constant overestimation of full-mouth CPITN-20.

### Recording sites with elevated plaque levels

The 12 tooth-surface combinations (o = oral, v = vestibular) ranking highest were 47-o, 37-o, 45-o, 44-o, 27-v, 17-v, 46-o, 34-o, 35-o, 36-o, 47-v, and 41-o (in order; Elevated-ref). For clinical practicability, 12 tooth-surface combinations 17-v, 27-v, 37-o, 36-o, 35-o, 34-o, 31-o, 41-o, 44-o, 45-o, 46-o, and 47-o (Elevated-12) and 6 tooth-surface combinations 17-v, 27-v, 36-o, 31-o, 41-o, and 46-o (Elevated-6) were evaluated (Fig. 2).

Across all plaque assessment methods (Table 2), the Elevated-12 subset showed a close correspondence with the data-driven Elevated-ref subset, with minimal deviations and consistently strong agreement. The Elevated-6 subset showed slightly reduced agreement but continued to track the reference satisfactorily across all indices. In contrast, CPITN subsets consistently showed weaker correspondence with the Elevated-ref subset and systematically lower plaque values, indicating underestimation of plaque-persistent sites.

**Table 2:** Performance of subsets representing elevated plaque levels relative to the elevated plaque level subset reference (Elevated-ref) across merged time points (T1–T3) for P%, TQHPI and RMNPI. RMSE: root-mean-square error, RMSE%: RMSE/Elevated-ref*100, ICC(2,1): two-way random-effects intraclass correlation for absolute agreement. The p value refers to the comparison of subsets to the Elevated-ref subset

	Mean ± SD	Bias	RMSE	RMSE%	ICC(2,1)	p value
**P%**						
Elevated-ref	33.1 ± 11.3					
Elevated-12	32.8 ± 10.9	−0.29	1.40	4.22	0.992	0.032
Elevated-6	30.7 ± 12.2	−2.44	5.09	15.36	0.908	< 0.001
CPITN-20	23.3 ± 9.7	−9.94	10.71	32.35	0.642	< 0.001
CPITN-12	18.8 ± 8.9	−14.35	15.40	46.51	0.425	< 0.001
**TQHPI**						
Elevated-ref	3.4 ± 0.6					
Elevated-12	3.4 ± 0.6	0.01	0.10	2.86	0.985	0.330
Elevated-6	3.3 ± 0.7	−0.07	0.32	9.51	0.866	0.022
CPITN-20	2.8 ± 0.6	−0.60	0.66	19.40	0.580	< 0.001
CPITN-12	2.6 ± 0.6	−0.82	0.91	26.82	0.391	< 0.001
**RMNPI**						
Elevated-ref	7.8 ± 0.8					
Elevated-12	7.8 ± 0.8	−0.01	0.12	1.61	0.989	0.847
Elevated-6	7.5 ± 0.9	−0.07	0.32	5.96	0.866	0.022
CPITN-20	6.8 ± 1.0	−0.80	0.95	12.55	0.625	< 0.001
CPITN-12	6.5 ± 1.1	−1.10	1.33	17.58	0.449	< 0.001

## Discussion

A central challenge for partial-mouth plaque recording is the distinct spatial heterogeneity of plaque distribution within the dentition. Previous studies have consistently shown that plaque is not evenly distributed across teeth or surfaces [7–9, 24, 25]. The heatmap visualisations in the present study confirm and extend these observations. Plaque coverage differed markedly between surfaces at baseline, with consistently elevated levels at mandibular oral and maxillary molar vestibular sites while maxillary oral surfaces showed lower and more homogeneous plaque accumulation. This spatial organisation is in particular consistent with the pattern described by Söder et al. [8], albeit at lower absolute plaque levels. Importantly, this same spatial ranking was preserved following plaque accumulation and after brushing, plaque reduction largely maintained the pre-existing distribution. From a methodological perspective, this temporal stability strengthens the plausibility that partial-mouth recording schemes can be designed to capture meaningful plaque information beyond a single cross-sectional snapshot.

The prevailing design of subsets for partial mouth recording reflects their historical development within periodontology (e.g. [19, 26].), where the tooth has traditionally served as the primary observational unit. In the context of periodontal disease, this focus is biologically plausible, as pathological changes such as pocket formation or attachment loss at individual sites ultimately affect the tooth as a functional entity. Consequently, subsets developed for periodontal research were optimised to characterise overall plaque presence at the tooth level rather than to resolve fine-grained, site-specific accumulation patterns. In contrast, the objectives of oral hygiene education and behavioural interventions differ fundamentally. From this perspective, plaque accumulation at specific tooth-surface locations is of primary relevance, as oral hygiene measures such as toothbrushing and interdental cleaning act locally and their effectiveness varies markedly between sites. This conceptual distinction may help explain why commonly used subsets, while valuable for periodontal epidemiology and clinical trials, appear comparatively coarse when applied to questions of oral hygiene performance, plaque dynamics, or site-specific educational feedback. In such contexts, indices that collapse surface-level information into tooth-level summaries may underestimate the heterogeneity of plaque accumulation and removal within the dentition. A surface-oriented approach to plaque assessment may therefore offer added value when the aim is not merely to describe disease-associated plaque burden, but to identify and monitor behaviourally relevant plaque retention sites.

Building on the conceptual distinction between tooth- and surface-oriented plaque assessment, the present study deliberately adopted a data-driven approach to partial-mouth recording. Rather than relying on predefined index teeth or historically established protocols, it was first sought to identify surface subsets that optimally represented full-mouth plaque coverage. Planimetric plaque assessment was used as the reference for this optimisation, as it provides an objective measure of plaque extent. On this basis, surface combinations of increasing size were systematically evaluated, resulting in optimised subsets comprising 12 and 6 surfaces, respectively. Notably, the 12-surface configuration is directly comparable in size to established tooth-based schemes that record six index teeth on two surfaces each (i.e., 12 tooth-surface combinations, as in the traditional Ramfjord approach). This provides a useful benchmark for interpreting the performance of the empirically optimised subset relative to familiar partial-mouth designs. In addition, the 6-surface subset represents a further condensation intended to maximise feasibility in time-constrained settings, providing an even more time-efficient subset for scenarios with restricted assessment time.

These optimised surface subsets demonstrated a high degree of representativeness for overall plaque levels. At the same time, the optimised surface subsets revealed practical limitations that are relevant for clinical and educational contexts as the data-driven selection resulted in an asymmetric distribution of surfaces across the dentition. While such asymmetry may be unproblematic in automated or semi-automated assessment pipelines, or in settings involving trained examiners, it may limit the feasibility and intuitive usability of these subsets in routine clinical practice. For this reason, the optimised 12- and 6-surface subsets were not intended as prescriptive clinical recording schemes, but rather as methodological benchmarks by approximating the best achievable performance of partial-mouth recording. Using these optimised subsets as comparators allows the performance of traditional tooth-based protocols and other surface selections to be interpreted relative to an empirically derived upper bound, rather than solely in relation to full-mouth recordings.

To contextualise the performance of the data-driven surface subsets, two established partial-mouth recording protocols were evaluated for comparison. These included the original Ramfjord index teeth, [5], a reduced Ramfjord subset derived through data-driven optimisation, and the two CPITN subsets comprising 20 and 12 tooth/surface combinations [27, 28], respectively. The original Ramfjord index teeth constitute one of the most influential partial recording schemes in dental research. Their selection reflects an effort to sample different regions of the dentition while maintaining feasibility. The CPITN subsets represent a different rationale for partial recording. Rather than focusing on a small number of index teeth, these protocols sample a larger portion of the dentition, thereby trading examination time for potentially improved representativeness.

Partial-mouth recording schemes for plaque assessment have been evaluated using single, index-based measures [19–23], rather than continuous surface-level metrics. In contrast, the present study provides a combined evaluation using planimetric plaque quantification alongside with two widely used plaque indices, enabling a comprehensive assessment across distinct measurement constructs. The inclusion of the TQHPI and RMNPI aligns with established oral hygiene research as systematic reviews of plaque control trials identify these indices among the most frequently applied outcomes in clinical studies of oral hygiene interventions (e.g. [29]). Notably, despite their widespread use, these indices have rarely been explicitly evaluated in the context of partial-mouth recording. Evaluating the subsets using both indices and planimetric plaque assessment therefore facilitates interpretation in the context of the existing literature while also testing whether subset performance is robust across conceptually different plaque measurement approaches.

The Optimised-12 subset reproduced full-mouth plaque levels with very high accuracy across all three plaque assessment approaches. Agreement with full-mouth measurements was near-perfect for planimetric plaque quantification and remained similarly strong for both plaque indices, with minimal bias and consistently high correlations. The Ramfjord-12 subset showed a slightly lower correspondence with full-mouth plaque levels, characterised by a small systematic underestimation. However, bias remained low and correlations with full-mouth values were high across planimetric assessment and both plaque indices. These findings are consistent with earlier evaluations of Ramfjord-type partial recording schemes using a range of plaque assessment methods, including the Silness and Löe Plaque Index [19, 22], the O’Leary Plaque Index [20], millimetre-based plaque measurements [21], and categorical plaque scoring based on crown coverage [23].

A comparable pattern was observed for the corresponding six-surface subsets, which exhibited a modest increase in variability but maintained low bias and strong correlations with full-mouth plaque measurements. Importantly, both the 12-surface and 6-surface subsets reliably reflected plaque accumulation following cessation of oral hygiene as well as plaque reduction after brushing, indicating that their performance was not limited to a single static condition. Taken together, these results suggest that not only the 12-surface subsets, but also reduced six-surface configurations, provide sufficiently accurate representations of full-mouth plaque levels to be considered for use in field or clinical studies or practice settings where examination time is constrained. However, some loss of spatial resolution is inevitable when plaque recording is substantially abbreviated. Localised changes at non-sampled sites may therefore go undetected, particularly when the aim is detailed site-specific feedback rather than overall monitoring.

The CPITN-20 subset systematically overestimated full-mouth plaque levels across all plaque assessment approaches. This overestimation was observed for planimetric plaque quantification as well as for both plaque indices and was accompanied by higher bias compared with the previously described subsets, despite generally high correlations with full-mouth values. In contrast, the CPITN-12 subset showed substantially lower bias and a closer agreement with full-mouth plaque levels, while maintaining similarly strong correlations. This finding is consistent with earlier studies using the Silness and Löe Plaque Index [19] and the O’Leary Plaque Index [20], which likewise reported better agreement with full-mouth recordings for CPITN-12 than for CPITN-20.

This pattern was consistent across oral hygiene states, with CPITN-20 overestimating plaque levels during baseline, plaque accumulation, and post-brushing conditions, whereas CPITN-12 provided a more balanced representation of both plaque accumulation and removal. Taken together, these results indicate that reducing the CPITN tooth set from 20 to 12 teeth improves agreement with full-mouth plaque measurements and yields performance characteristics more comparable to those of the surface-optimised and Ramfjord-based subsets.

The divergent performance of CPITN subsets can be explained by the specific tooth-surface combinations they include. CPITN sampling emphasises oral and vestibular surfaces of molars across quadrants and adds incisor sites. When estimating overall full-mouth plaque levels, this scheme tends to overestimate because molar surfaces generally carry higher plaque levels than the dentition average; relative underrepresentation of low-plaque anterior and vestibular sites further shifts the mean upward.

In addition to subsets optimised to represent overall plaque levels, subsets focusing on tooth-surface combinations with elevated plaque levels were evaluated to assess whether partial-mouth recording schemes can adequately capture regions that disproportionately contribute to plaque burden. Such subsets are of particular relevance for oral hygiene research and education, where identifying and monitoring plaque-prone locations is often more informative than estimating average plaque levels alone.

The empirically derived elevated-plaque reference subset revealed a distinct spatial pattern characterised by a concentration of plaque at mandibular oral and posterior sites. Notably, the tooth-surface combinations identified as exhibiting the highest plaque levels showed a striking correspondence with those reported by Söder et al. [8], who described a nearly identical distribution of plaque-prone sites in adults. This close agreement provides external support for the robustness of the observed elevated-plaque pattern and suggests that these sites reflect stable features of plaque distribution rather than cohort-specific artefacts.

Based on this reference, reduced elevated-plaque subsets comprising 12 and 6 tooth–surface combinations were evaluated. For clinical utility, the empirically identified elevated-plaque reference set was slightly adapted, resulting in subsets that consistently included the vestibular surfaces of both second molars, while the remaining sites comprised a varying number of oral surfaces depending on whether the 12- or 6-surface configuration was applied. Both subsets captured elevated plaque levels with low bias and high correlations relative to the reference set, although, as expected, the six-surface subset showed slightly increased variability. Importantly, both configurations preserved the relative ranking of elevated plaque levels across oral hygiene states, indicating that they were suitable for monitoring plaque accumulation and removal at plaque-prone sites. In contrast, CPITN subsets, included here as molar-weighted tooth sets likely to capture high plaque burden, showed poorer agreement when benchmarked against the elevated-plaque reference. Both CPITN-20 and CPITN-12 subsets systematically underestimated plaque levels relative to elevated-plaque sites, with larger bias compared with the empirically derived subsets. This underestimation was consistent across baseline, plaque accumulation, and post-brushing conditions, indicating that broad tooth-based sampling schemes may dilute information from plaque-prone surfaces when the outcome of interest is concentrated at specific locations.

Several limitations of the present study should be acknowledged. First, the empirically derived subsets were identified and evaluated within a single study population and setting. Although the spatial plaque distribution patterns observed here were consistent with earlier reports, including classic distribution studies, the exact composition of optimal tooth-surface subsets may vary across populations with different age distributions, oral hygiene behaviours, or dental status. External validation in independent cohorts will therefore be important to assess the transportability of the proposed subsets. Second, the present analysis focused on the ability of partial-mouth recording schemes to represent plaque levels and their short-term changes, rather than on their capacity to predict clinical outcomes. Whether the proposed subsets are suitable for risk stratification or prediction of plaque-associated diseases, such as caries or periodontitis, remains to be investigated in longitudinal studies linking partial-mouth plaque measures to disease incidence or progression.

Finally, plaque accumulation and removal were assessed over a short observation period under controlled conditions. The performance of the proposed subsets in longer-term monitoring or under routine clinical care conditions warrants further study.

## Conclusion

In conclusion, this study provides a comprehensive evaluation of partial-mouth plaque recording using three complementary measurement approaches (planimetric plaque quantification, TQHPI and RMNPI) across baseline conditions as well as plaque accumulation and post-brushing states. For estimating overall plaque levels, the established CPITN-12 and Ramfjord-12 subsets approximated full-mouth measurements closely, whereas CPITN-20 showed systematic overestimation and appears less suitable for this purpose.

Beyond these established protocols, the present work introduces two novel, empirically derived recording schemes. First, a reduced Ramfjord-6 subset was derived from the original Ramfjord surface set and retained favourable agreement with full-mouth plaque levels while further reducing examination effort, offering a time-efficient alternative for constrained study or clinical settings. Second, new surface-based subsets were developed to capture tooth-surface combinations with elevated plaque levels, with both 12- and 6-surface configurations providing robust performance for monitoring plaque-prone sites during plaque accumulation and following brushing.

## Supplementary Information

Below is the link to the electronic supplementary material.

Supplementary file1 (DOCX 23 KB)

## Data Availability

All data supporting the findings of this study are available within the paper and its Supplementary Information.
