# Supplementary material for: Partial-mouth plaque recording revisited: evaluation of tooth- and surface-based subsets using a data-driven benchmark
Source: Clin Oral Investig. 2026 Jul 16;30(8):332. doi: 10.1007/s00784-026-07024-1 (PMC13375822; doi:10.1007/s00784-026-07024-1)
Supplement: Supplementary file 1 — Supplementary file1 (DOCX 23 KB) [file 784_2026_7024_MOESM1_ESM.docx]

Table S1 Performance of subsets relative to full-mouth plaque assessments across the time points T1, T2 and T3 for P%, TQHPI and RMNPI. RMSE: root-mean-square error, RMSE%: percentage of the mean full-mouth score (RMSE/full-mouth*100), ICC(2,1): two-way random-effects intraclass correlation for absolute agreement . The p value refers to the comparison of subsets to the full mouth plaque level.

|  | **Mean** | **Bias** | **RMSE** | **RMSE%** | **ICC** | **Wilcoxon** |
| --- | --- | --- | --- | --- | --- | --- |
| **P% T1** | | | | | | |
| Full-mouth | 16.0 ± 5.5 |  |  |  |  |  |
| Optimised-12 | 16.1 ± 6.1 | 0.12 | 1.84 | 11.49 | 0.950 | 0.761 |
| Ramfjord-12 | 15.0 ± 6.0 | -1.00 | 1.79 | 11.21 | 0.952 | 0.001 |
| Optimised-6 | 15.2 ± 6.3 | -0.79 | 2.19 | 13.72 | 0.931 | 0.021 |
| Ramfjord-6 | 14.9 ± 6.4 | -1.06 | 2.17 | 13.55 | 0.935 | 0.004 |
| CPITN-20 | 19.8 ± 7.6 | 3.78 | 4.81 | 30.11 | 0.774 | < 0.001 |
| CPITN-12 | 15.9 ± 6.9 | -0.10 | 2.39 | 14.97 | 0.926 | 0.416 |
| **P% T2** | | | | | | |
| Full-mouth | 25.9 ± 8.4 |  |  |  |  |  |
| Optimised-12 | 25.3 ± 8.1 | -0.57 | 2.92 | 11.28 | 0.937 | 0.516 |
| Ramfjord-12 | 24.5 ± 8.3 | -1.46 | 2.35 | 9.09 | 0.975 | < 0.001 |
| Optimised-6 | 25.7 ± 8.8 | -0.21 | 2.94 | 11.34 | 0.961 | 0.952 |
| Ramfjord-6 | 25.7 ± 8.8 | -0.18 | 3.37 | 13.01 | 0.935 | 0.887 |
| CPITN-20 | 30.8 ± 9.2 | 4.87 | 6.10 | 23.54 | 0.792 | < 0.001 |
| CPITN-12 | 26.0 ± 8.9 | 0.06 | 2.06 | 7.95 | 0.972 | 0.808 |
| **P% T3** | | | | | | |
| Full-mouth | 14.6 ± 5.1 |  |  |  |  |  |
| Optimised-12 | 15.1 ± 5.6 | 0.53 | 1.78 | 12.19 | 0.946 | 0.119 |
| Ramfjord-12 | 13.4 ± 4.8 | -1.21 | 1.83 | 12.53 | 0.935 | < 0.001 |
| Optimised-6 | 14.1 ± 5.3 | -0.48 | 1.98 | 13.55 | 0.929 | 0.177 |
| Ramfjord-6 | 13.6 ± 5.1 | -1.03 | 1.97 | 13.51 | 0.923 | 0.004 |
| CPITN-20 | 19.0 ± 7.6 | 4.36 | 5.18 | 35.45 | 0.738 | < 0.001 |
| CPITN-12 | 14.4 ± 6.0 | -0.16 | 2.09 | 14.34 | 0.930 | 0.253 |
| **TQHPI T1** | | | | | | |
| Full-mouth | 2.4 ± 0.4 |  |  |  |  |  |
| Optimised-12 | 2.4 ± 0.5 | 0.02 | 0.20 | 8.53 | 0.900 | 0.579 |
| Ramfjord-12 | 2.2 ± 0.5 | -0.19 | 0.30 | 12.63 | 0.814 | 0.001 |
| Optimised-6 | 2.3 ± 0.5 | -0.08 | 0.24 | 9.91 | 0.872 | 0.079 |
| Ramfjord-6 | 2.3 ± 0.6 | -0.12 | 0.41 | 17.23 | 0.715 | 0.123 |
| CPITN-20 | 2.6 ± 0.5 | 0.20 | 0.29 | 12.15 | 0.838 | < 0.001 |
| CPITN-12 | 2.4 ± 0.5 | -0.03 | 0.25 | 10.26 | 0.880 | 0.469 |
| **TQHPI T2** | | | | | | |
| Full-mouth | 3.1 ± 0.5 |  |  |  |  |  |
| Optimised-12 | 3.0 ± 0.5 | -0.09 | 0.20 | 6.48 | 0.917 | 0.010 |
| Ramfjord-12 | 2.9 ± 0.5 | -0.15 | 0.22 | 7.28 | 0.903 | < 0.001 |
| Optimised-6 | 3.0 ± 0.5 | -0.07 | 0.23 | 7.45 | 0.898 | 0.166 |
| Ramfjord-6 | 3.0 ± 0.5 | -0.04 | 0.33 | 10.64 | 0.792 | 0.829 |
| CPITN-20 | 3.2 ± 0.5 | 0.17 | 0.28 | 9.31 | 0.835 | 0.002 |
| CPITN-12 | 3.1 ± 0.5 | -0.01 | 0.19 | 6.10 | 0.922 | 0.894 |
| **TQHPI T3** | | | | | | |
| Full-mouth | 2.2 ± 0.4 |  |  |  |  |  |
| Optimised-12 | 2.3 ± 0.5 | 0.03 | 0.20 | 9.04 | 0.906 | 0.459 |
| Ramfjord-12 | 2.1 ± 0.5 | -0.16 | 0.26 | 11.77 | 0.822 | < 0.001 |
| Optimised-6 | 2.1 ± 0.6 | -0.10 | 0.29 | 12.90 | 0.827 | 0.144 |
| Ramfjord-6 | 2.1 ± 0.6 | -0.15 | 0.36 | 16.29 | 0.729 | 0.016 |
| CPITN-20 | 2.5 ± 0.5 | 0.28 | 0.34 | 15.41 | 0.762 | < 0.001 |
| CPITN-12 | 2.3 ± 0.5 | 0.03 | 0.18 | 7.98 | 0.916 | 0.600 |
| **RMNPI T1** | | | | | | |
| Full-mouth | 6.0 ± 1.0 |  |  |  |  |  |
| Optimised-12 | 6.1 ± 1.0 | 0.06 | 0.48 | 7.93 | 0.874 | 0.622 |
| Ramfjord-12 | 6.0 ± 1.0 | -0.05 | 0.55 | 9.10 | 0.841 | 0.393 |
| Optimised-6 | 5.8 ± 1.1 | -0.18 | 0.52 | 8.63 | 0.872 | 0.075 |
| Ramfjord-6 | 5.9 ± 1.1 | -0.08 | 0.72 | 12.01 | 0.749 | 0.304 |
| CPITN-20 | 6.5 ± 1.0 | 0.49 | 0.62 | 10.23 | 0.822 | < 0.001 |
| CPITN-12 | 6.2 ± 1.1 | 0.16 | 0.44 | 7.29 | 0.907 | 0.027 |
| **RMNPI T2** | | | | | | |
| Full-mouth | 7.1 ± 0.8 |  |  |  |  |  |
| Optimised-12 | 7.0 ± 0.8 | -0.17 | 0.39 | 5.48 | 0.882 | 0.014 |
| Ramfjord-12 | 7.1 ± 0.9 | -0.09 | 0.45 | 6.23 | 0.863 | 0.106 |
| Optimised-6 | 6.9 ± 0.9 | -0.21 | 0.45 | 6.32 | 0.854 | 0.008 |
| Ramfjord-6 | 7.0 ± 1.0 | -0.15 | 0.64 | 8.97 | 0.743 | 0.136 |
| CPITN-20 | 7.4 ± 0.8 | 0.27 | 0.43 | 5.96 | 0.861 | 0.001 |
| CPITN-12 | 7.2 ± 0.9 | 0.06 | 0.33 | 4.67 | 0.922 | 0.329 |
| **RMNPI T3** | | | | | | |
| Full-mouth | 5.7 ± 0.9 |  |  |  |  |  |
| Optimised-12 | 5.8 ± 1.1 | 0.09 | 0.41 | 7.10 | 0.919 | 0.131 |
| Ramfjord-12 | 5.7 ± 1.0 | -0.02 | 0.51 | 8.84 | 0.856 | 0.399 |
| Optimised-6 | 5.5 ± 1.2 | -0.20 | 0.52 | 9.03 | 0.878 | 0.064 |
| Ramfjord-6 | 5.7 ± 1.2 | -0.08 | 0.73 | 12.72 | 0.751 | 0.388 |
| CPITN-20 | 6.4 ± 1.0 | 0.67 | 0.73 | 12.78 | 0.768 | < 0.001 |
| CPITN-12 | 6.0 ± 1.1 | 0.29 | 0.59 | 10.29 | 0.835 | 0.007 |
